# Rhombencephalosynapsis: A Rare Hindbrain Malformation

**DOI:** 10.7759/cureus.65400

**Published:** 2024-07-26

**Authors:** Sanjay M Khaladkar, Neeha A Jhala, Amanya Shukla, Rohan Shah, Eshan Chetan Durgi

**Affiliations:** 1 Radiodiagnosis, Dr. D. Y. Patil Medical College, Hospital & Research Centre, Dr. D. Y. Patil Vidyapeeth, Pune, IND

**Keywords:** hind brain, ataxia, cerebellum, cerebellar vermis, rhombencephalon

## Abstract

Rhombencephalosynapsis (RES) is a rare condition found in the pediatric population. It occurs due to a fundamental failure of vermian differentiation caused by faulty gene expression in the earliest patterning areas of the brain between days 28 and 41 of gestation, resulting in a fused cerebellum. This report aims to discuss cases of this rare hindbrain malformation, identify its features on MRI, diagnose any associated anomalies, classify it based on severity, and study its syndromic associations. We report two rare cases of RES in patients presenting with complaints of ataxia, global motor developmental delay, hypotonia, and dysarthria, who underwent an MRI of the brain.

## Introduction

Rhombencephalosynapsis (RES), termed thus by Gross and Hoff in 1959 [1], was first described by Obersteiner in 1914 during an incidental postmortem examination of a 28-year-old male who had committed suicide [2]. It was initially diagnosed using MRI in 1991 [3]. The prevalence of RES is 1 per 1,000,000, with a frequency of 0.13% in the pediatric population [4]. No specific chromosomal abnormality, metabolic factor, or teratogenic factor has been identified as a cause of this condition. However, a potential link with autosomal recessive inheritance has been suggested [5]. It typically occurs sporadically with no familial recurrence [6].

The cerebellar vermis is a crucial structure connecting the two hemispheres of the cerebellum and serves as a central unpaired midline structure. It plays a pivotal role in coordinating body movements, speech, eye movements, maintaining equilibrium, and regulating emotions. Embryologically, RES arises from a fundamental failure of vermian differentiation due to faulty gene expression in the earliest patterning areas of the brain between days 28 and 41 of gestation, resulting in a fused cerebellum. Clinically, patients present with cerebellar symptoms such as ataxia, global motor developmental delay, hypotonia, and dysarthria [7]. Given that clinical features overlap with those of other hindbrain malformations, MRI is the preferred modality for diagnosing the condition. Classified into different patterns based on its severity, RES can manifest as an isolated malformation of posterior fossa structures or in conjunction with other abnormalities of the central nervous system (CNS) or systemic anomalies, termed RES "plus" [1]. We present two cases of RES and highlight the importance of diagnostic imaging in the clinico-radiological diagnosis of the condition.

## Case presentation

Case 1

A one-year-old first-born male child presented with delayed motor developmental milestones and ataxia. The patient was able to crawl, and neck-holding was present. He had been born to consanguineous parents, delivered prematurely at six months via normal vaginal delivery, weighing 1 kg, and had cried immediately after birth. He had spent one month in the neonatal intensive care unit (NICU) due to distress. The mother had a healthy pregnancy with no history of drug intake. Antenatal ultrasound showed no anomalies. MRI brain was performed due to ataxia.

MRI Findings

**Figure 1:**
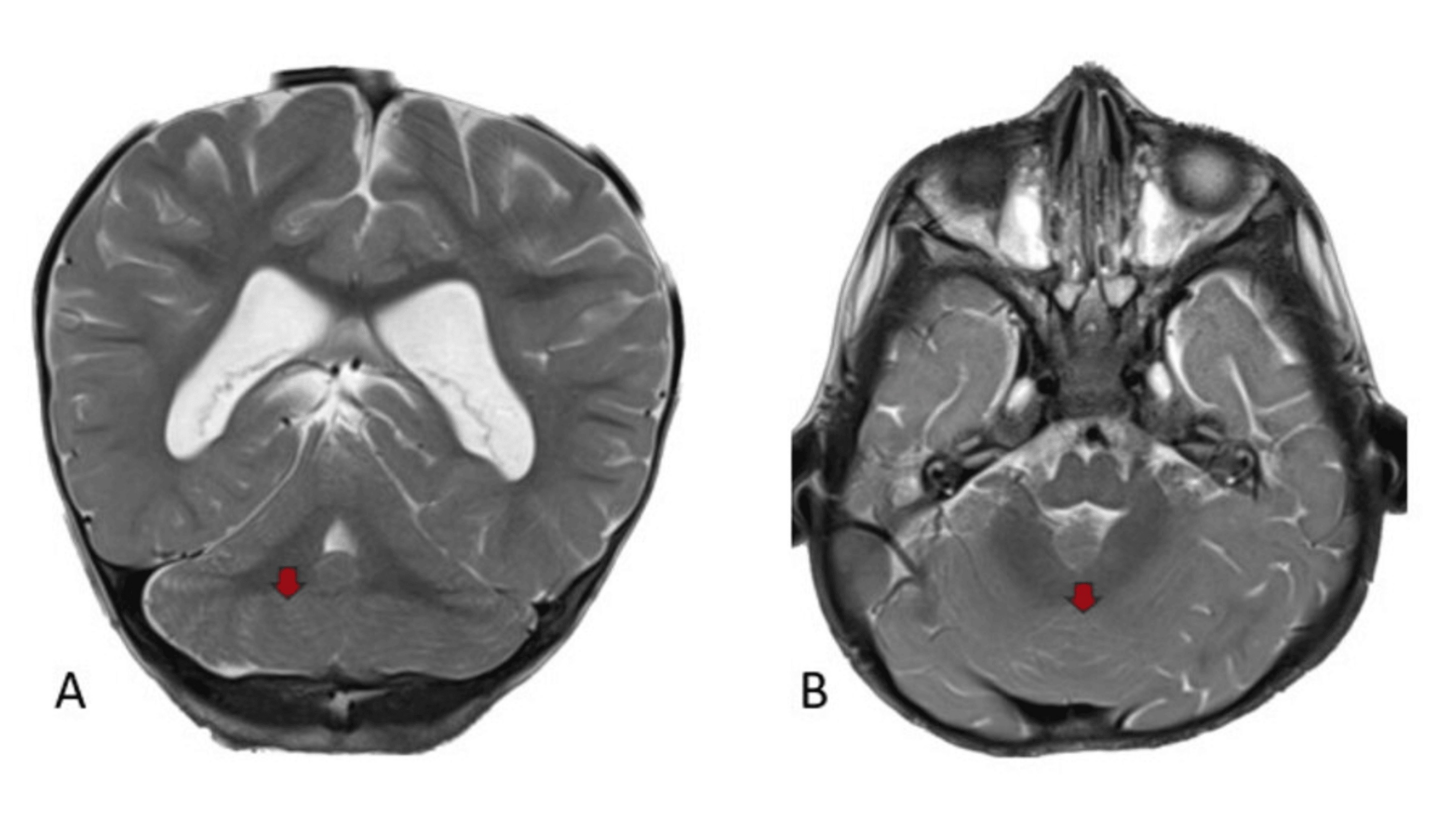
MRI T2WI findings A (coronal) and B (axial) views reveal uninterrupted continuity of white matter and folia across the midline (red arrows), indicative of a single-lobed cerebellum with transversely oriented folia, confirming fusion MRI: magnetic resonance imaging; T2WI: T2-weighted imaging

On T2-weighted imaging (T2WI), there was an absence of the cerebellar vermis and primary fissure, with the fusion of both cerebellar hemispheres and dentate nuclei, consistent with RES. Mild dilatation of the third ventricle and both lateral ventricles was also noted. The rest of the supratentorial compartment appeared normal. Figure 1 depicts MRI T2WI images indicative of a single-lobed cerebellum with transversely oriented folia, confirming cerebellar fusion. 

**Figure 2:**
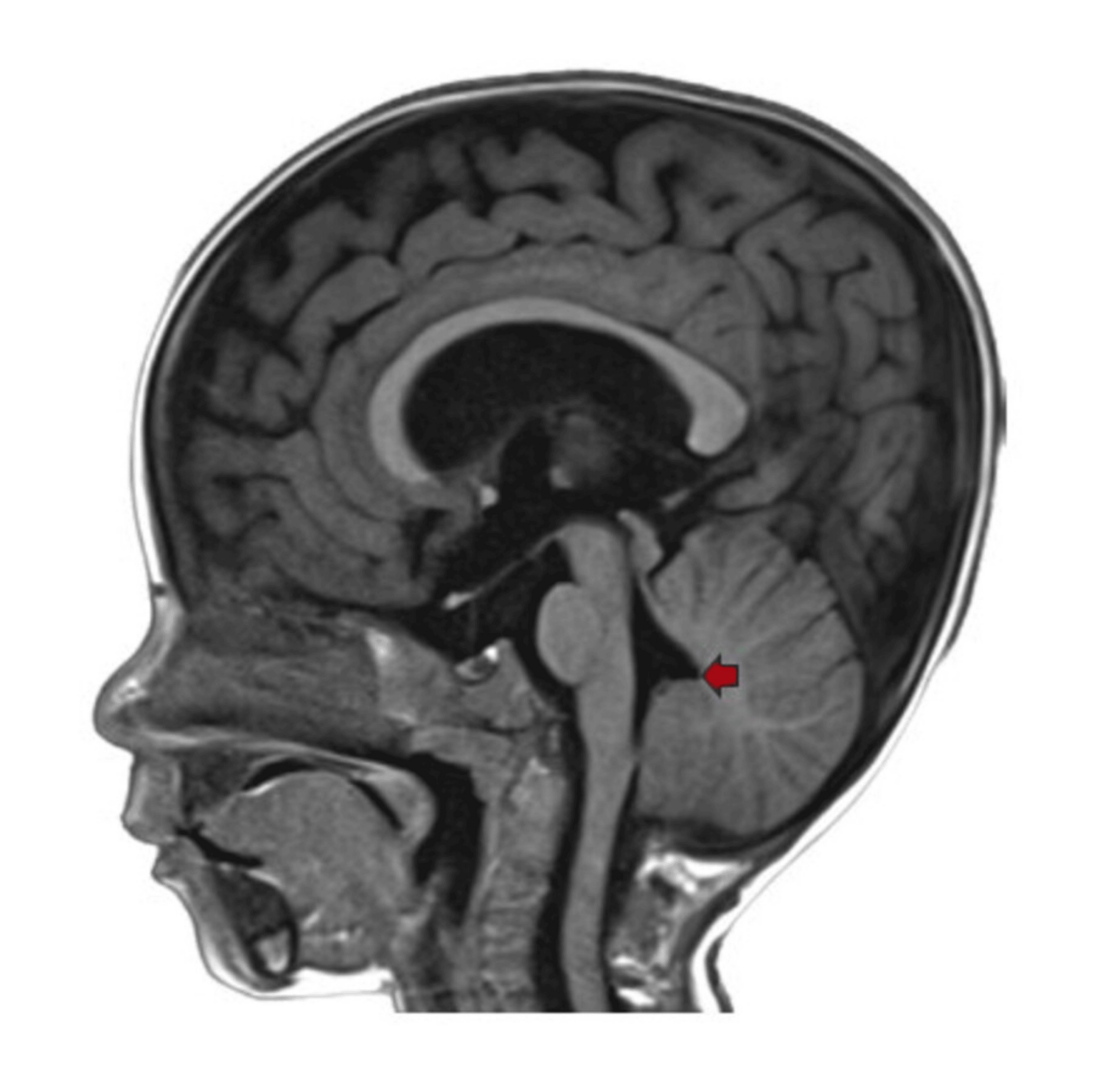
MRI T1WI sagittal image The image depicts the absence of the primary fissure and rounding of the fastigial recess (red arrow) MRI: magnetic resonance imaging; T1WI: T1-weighted imaging

Figure 2 depicts a T1WI MRI image showing the absence of the primary fissure and rounding of the fastigial recess. 

Case 2

A three-year-old first-born male child presented with delayed motor developmental milestones, dysarthria, and ataxia. He had been born to consanguineous parents via normal vaginal delivery at full term, weighing 2.8 kg. The baby had cried within five minutes after birth, and there was no history of NICU admission. The mother did not report any drug intake during the antenatal period. An anomaly scan performed at 18 weeks gestation was normal. Clinically, the patient exhibited frontal bossing, with the anteroposterior diameter of the skull being greater than the transverse diameter. MRI brain was performed due to ataxia.

MRI Findings

On T2WI, there was an absence of the cerebellar vermis and primary fissure, along with the fusion of both cerebellar hemispheres and dentate nuclei, indicative of RES. The rostrum, body, and splenium of the corpus callosum were not visualized, with only a small part of the genu visible. The head appeared elongated with an increased anteroposterior dimension compared to width, suggestive of dolichocephaly. Asymmetrical dilation of the bilateral lateral ventricles was observed, with the right ventricle more dilated than the left. Axial images showed parallel orientation of the lateral ventricles. Additionally, a high-riding third ventricle and an opening in the interhemispheric fissure were noted. A dorsal interhemispheric cyst communicated with the third ventricle and the lateral ventricles. There was posteromedial herniation of the bilateral medial temporal lobes, with dilated temporal horns projecting into the ambient and supra cerebellar cisterns.

**Figure 3:**
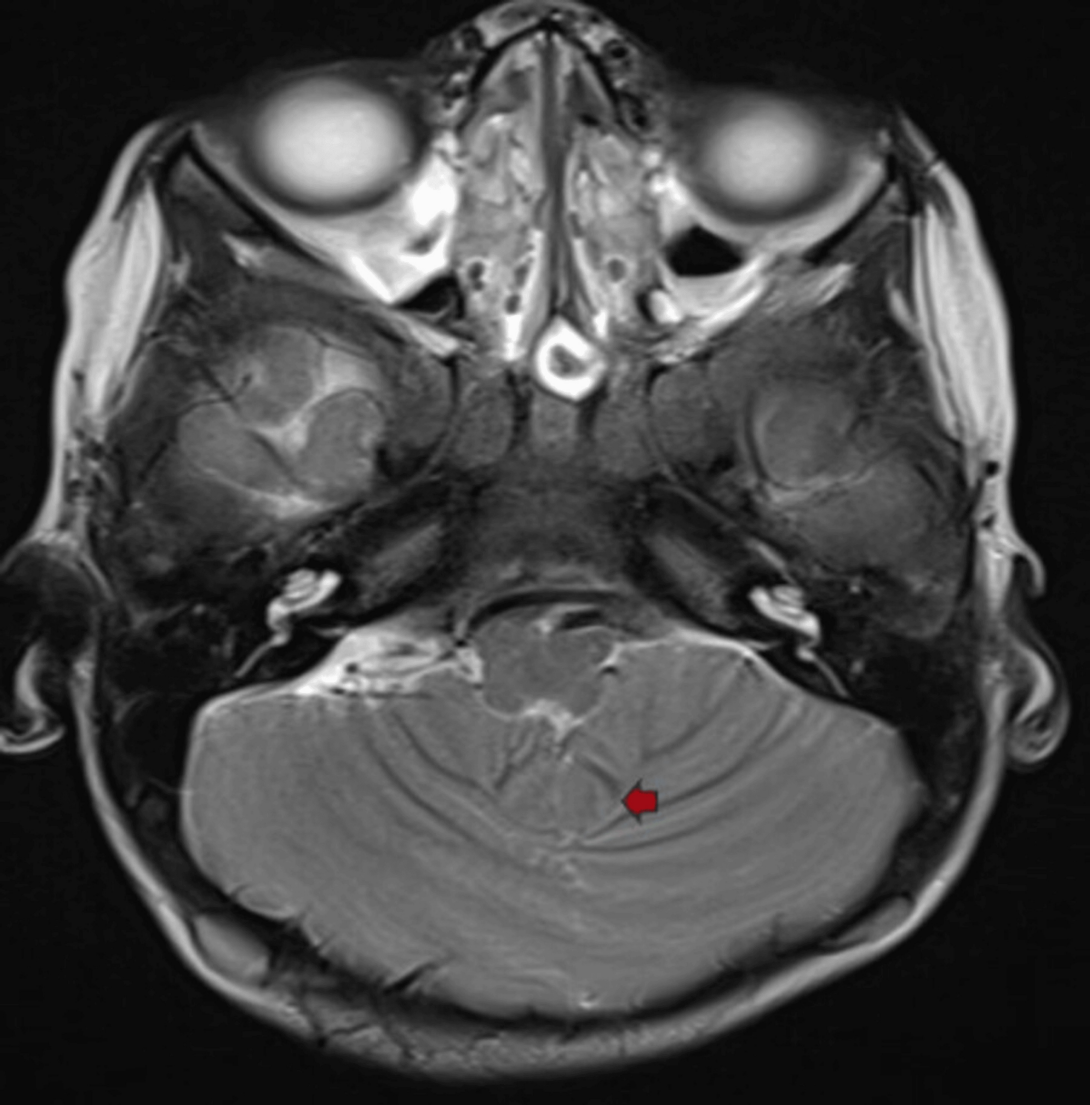
MRI axial T2WI findings The image shows a single-lobe cerebellum with vermian hypoplasia (red arrow) MRI: magnetic resonance imaging; T2WI: T2-weighted imaging

Figure 3 shows the MRI T2WI image depicting a single-lobe cerebellum with vermian hypoplasia.

**Figure 4:**
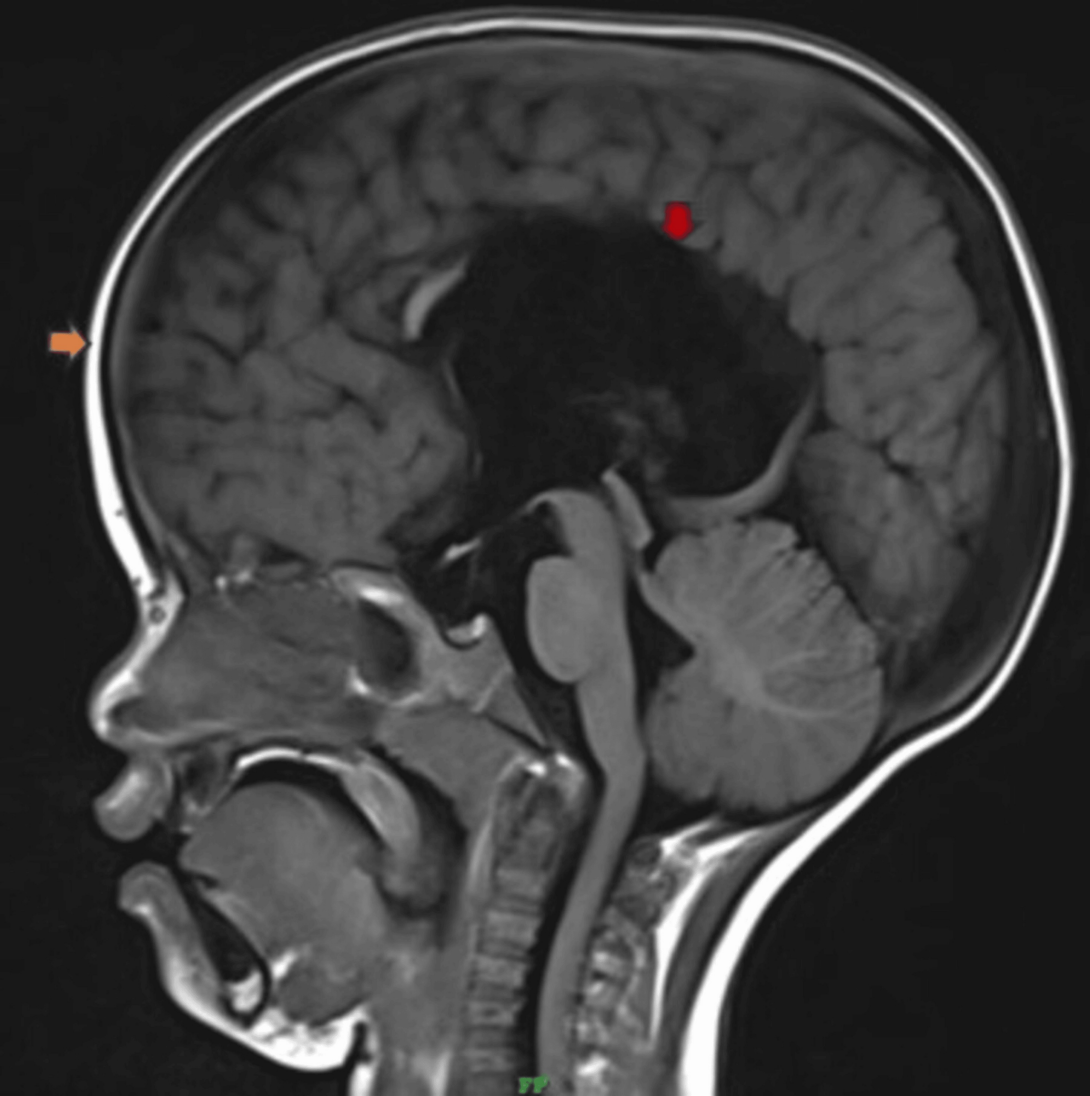
MRI T1WI sagittal image The image demonstrates partial dysgenesis of the corpus callosum (red arrow) and dolichocephaly (yellow arrow) MRI: magnetic resonance imaging; T1WI: T1-weighted imaging

 Figure 4 depicts the MRI T1WI image demonstrating partial dysgenesis of the corpus callosum and dolichocephaly.

**Figure 5:**
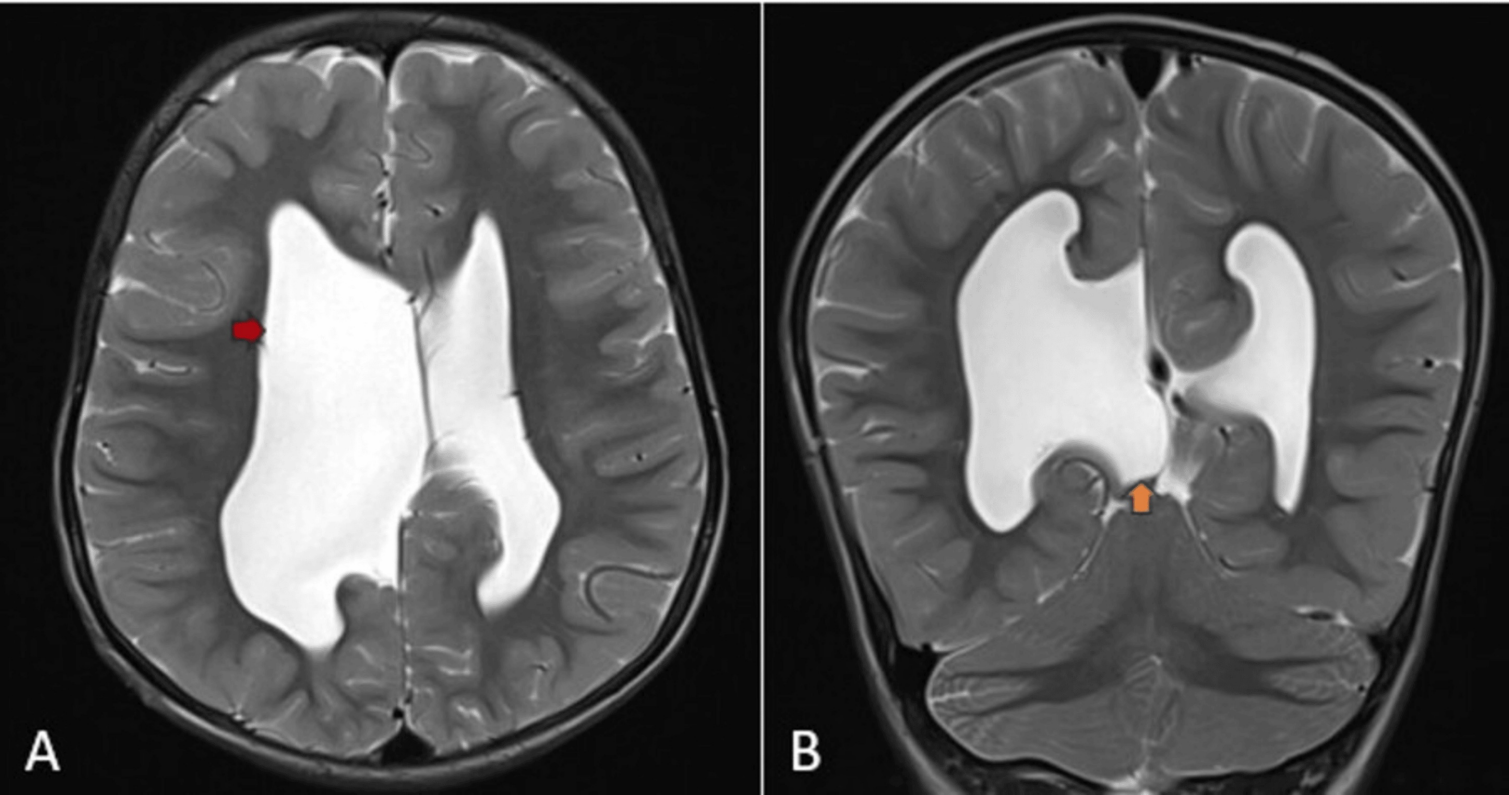
MRI T2WI images Image A (axial) shows asymmetrical dilatation of the lateral ventricles (red arrow). Image B (coronal) demonstrates a dorsal interhemispheric cyst (yellow arrow) MRI: magnetic resonance imaging; T2WI: T2-weighted imaging

Figure 5 depicts MRI T2WI images showing asymmetrical dilatation of the lateral ventricles along with a dorsal interhemispheric cyst.

Based on the findings above, Case 1 was diagnosed with RES with mild non-obstructive hydrocephalus. The clinician advised the patient to undergo physical therapy for delayed motor developmental milestones and movement abnormalities. Case 2 was diagnosed with RES with partial corpus callosal dysgenesis, non-obstructive hydrocephalus, dorsal interhemispheric cyst, and dolichocephaly. Ventriculoperitoneal shunt placement was planned for hydrocephalus. On follow-up, the obstructive symptoms of the patients had improved. The patient was also undergoing physical therapy for delayed motor developmental milestones and dysarthria.

## Discussion

The cerebellar vermis is a crucial structure that connects the two hemispheres of the cerebellum and serves as a central unpaired midline structure. In midline sagittal images, it displays multiple lobules with a distinctive folial pattern. Functionally, the vermis integrates sensory inputs from the spinal cord, cerebrum, and cerebellum, acting as a vital relay center. It plays a pivotal role in coordinating body movements, speech, eye movements, maintaining equilibrium, and regulating emotions [7]. Pathology or absence of the vermis can result in disorders related to movement and balance and emotional disturbances. The normal function of the cerebellar-thalamo-prefrontal circuit involves controlling motor skills, inhibitory functions, and executive functions. Therefore, the absence of the vermis can disrupt these essential cognitive and motor functions [7].

The cerebellar vermis, situated along the midline, is anatomically subdivided based on its location relative to key landmarks. The region posterior or caudal to the principal fissure is termed the posterior vermis, while the area anterior or rostral to this fissure is known as the anterior vermis. Additionally, the tissue immediately caudal to the fastigium is identified as the nodulus [8]. In RES, there is a continuity of folia and white matter across the midline without a transition to the vermis [7]. Multiple theories and hypotheses have been proposed regarding the occurrence of RES [1]. The most widely accepted hypothesis attributes it to a primary failure of vermian differentiation caused by defective gene expression in the early patterning centers of the brain. This results in an undivided cerebellum with fused cerebellar hemispheres, rather than merely a maldevelopment of the vermis [1]. On gross pathology, both cerebellar hemispheres appear fused in the midline, displaying transversely oriented folia (single-lobed cerebellum), with no intervening cyst. The cerebellar vermis is either poorly differentiated or entirely absent [7].

Distinctive features on MRI in cases of RES include a continuous white matter of the folia across the midline of the cerebellum, which does not transition to the vermis. The folia appears transversely oriented, indicating a single-lobed cerebellum. On axial sections, the fourth ventricle exhibits a keyhole- or diamond-shaped appearance instead of the typical crescent shape. This anomaly is attributed to vermian agenesis, the fusion of dentate nuclei, and the positioning of the middle cerebellar peduncles behind the pointed fourth ventricle. Additionally, sagittal images reveal the absence of the primary fissure and a rounded rather than sharp fastigial recess of the fourth ventricle [7].

Based on the few reported cases of both isolated RES and RES with associated anomalies, the features have been classified into five distinct patterns depending on the severity of the condition: (1) Complete: this pattern involves the absence of all parts of the vermis; (2) Partial severe: here, both the posterior and anterior vermis are absent, though a partially developed nodulus may be visible; (3) Partial moderate: this form is characterized by the absence of the posterior vermis, with varying degrees of deficiency in the anterior vermis and nodulus; (4) Partial mild: in this pattern, there is a fusion of the central vermis, with some anterior vermis above the fusion and posterior vermis and nodulus below; and (5) Atypical: this pattern shows the absence of the posterior vermis and nodulus, with remnants of the vermis still discernible [8].

In our cases, Case 1 was categorized as complete RES with the absence of all parts of the vermis, and Case 2 was categorized as partial mild RES with vermian hypoplasia. RES may manifest either alone as a malformation of the posterior fossa structures or in combination with additional defects in the CNS and/or systemic abnormalities, referred to as RES "plus" [1]. RES can be associated with other CNS anomalies such as ventriculomegaly, hydrocephalus, absent/rudimentary/dysgenic corpus callosum, absent septum pellucidum/fused thalami, abnormalities in the tectum and fornices, hypoplasia of the temporal lobes and olivary nuclei, anterior commissure and optic chiasma, agenesis of the posterior lobe of the pituitary, hippocampal hypoplasia, supra-tentorial clefts, and cranial synostosis [1].

Both of our cases are categorized as RES 'plus' disorders and have been associated with CNS anomalies such as ventriculomegaly, partial corpus callosal dysgenesis, dolichocephaly, and hydrocephalus. Associated systemic anomalies include cardiovascular, respiratory, urinary system, and skeletal abnormalities such as scoliosis, segmentation anomalies in the spine, phalangeal hypoplasia, duplication of thumbs, polydactyly, hexadactyly, and genu valgum [1]. RES can also be a component of syndromes such as VACTERL (Vertebral anomalies, anal atresia, cardiovascular defects, esophageal atresia, T-O fistula, renal and limb/radial anomalies), VACTERL-H (VACTERL anomalies with hydrocephalus), Gomez-Lopez-Hernandez syndrome (GLHS), and cerebello-trigeminal-dermal dysplasias [1].

RES and Joubert syndrome (Table 1) must be differentiated from each other, as both are rare congenital hindbrain anomalies that share characteristics such as agenesis or hypogenesis of the cerebellar vermis [9]. Our cases demonstrated features of RES, including a single-lobed cerebellum, abnormal transverse orientation of cerebellar folia, teardrop-shaped fourth ventricle, and hydrocephalus, along with supratentorial anomalies like corpus callosal dysgenesis.

**Table 1:** Features differentiating rhombencephalosynapsis from Joubert syndrome

Features	Rhombencephalosynapsis	Joubert syndrome
Presenting age	Variable to late	Early
Vermian agenesis/hypogenesis	No vermian cleft (single-lobed cerebellum)	Midline cleft present (apposition of hemispheres in midline)
Vermian folia	Present with abnormal transverse orientation	Total or near total absence of folia
Dentate nuclei	Fused across midline	Dysplastic - fragmented - heterotopic
Superior cerebellar peduncles	Fused	Thickened and elongated with molar tooth sign
Inferior colliculi	Fused	Normal
Abnormal fourth ventricle	Heart-/teardrop-shaped	Triangular/batwing-shaped
Aqueductal stenosis	May be present	Not present
Hydrocephalus and supra tentorial anomalies	Usually present	Rare to absent
Prognosis	Variable	Poor

## Conclusions

RES occurs sporadically and is classified into partial or complete forms, encompassing five distinct patterns. Absence or pathology of the vermis can lead to movement and equilibrium disorders, along with emotional disturbances. To sum up, RES presents a rare but significant challenge in neurodevelopmental diagnostics. Absence or pathology of the vermis can lead to movement and equilibrium disorders, along with emotional disturbances. With increasing awareness of this condition and improved availability and utility of MRI, early diagnosis is crucial for guiding surgical or supportive management strategies aimed at enhancing patients' quality of life. Our cases illustrate typical RES features and highlight the role of MRI in diagnosis and management. Understanding its varied presentations and associated anomalies is crucial for effective clinical management and further research in this field.
